# Temperature Dependent Mechanical Property of PZT Film: An Investigation by Nanoindentation

**DOI:** 10.1371/journal.pone.0116478

**Published:** 2015-03-13

**Authors:** Yingwei Li, Shangming Feng, Wenping Wu, Faxin Li

**Affiliations:** 1 Department of Engineering mechanics, School of Civil Engineering, Wuhan University, Wuhan, China; 2 State Key Laboratory of Water Resources and Hydropower Engineering Science, Wuhan University, Wuhan, China; 3 State Key Lab for Turbulence and Complex Systems, College of Engineering, Peking University, Beijing, China; University of Akron, UNITED STATES

## Abstract

Load-depth curves of an unpoled Lead Zirconate Titanate (PZT) film composite as a function of temperature were measured by nanoindentation technique. Its reduce modulus and hardness were calculated by the typical Oliver-Pharr method. Then the true modulus and hardness of the PZT film were assessed by decoupling the influence of substrate using methods proposed by Zhou et al. and Korsunsky et al., respectively. Results show that the indentation depth and modulus increase, but the hardness decreases at elevated temperature. The increasing of indentation depth and the decreasing of hardness are thought to be caused by the decreasing of the critical stress needed to excite dislocation initiation at high temperature. The increasing of true modulus is attributed to the reducing of recoverable indentation depth induced by back-switched domains. The influence of residual stress on the indentation behavior of PZT film composite was also investigated by measuring its load-depth curves with pre-load strains.

## Introduction

Lead titanate zirconate (PZT) films have been integrated in microelectromechanical systems (MEMS) working as sensors, transducers and actuators et al. due to their extraordinary electromechanical coupling property [1–3]. Because PZT films were usually experienced to concentrated electric field during working, their polarization response at nanoscale has got extensive researches by using Piezoelectric Force Microscopy (PFM) [4]. Meanwhile, select works concerning its mechanical property were also conducted [5–9], as understanding the mechanical property of PZT films is very important for the design and the lifetime assessment of PZT film devices [10,11]. For instance, the electromechanical coefficients of PZT films such as piezoelectric constant (d_33_ and d_31_) depended on their mechanical property [10,12], and these are the key parameters for designing PZT film devices [3].

Most of the reports concerning the mechanical characteristics of PZT films were conducted by nanoindentation technique [5–9], as it has high sensitivity [13] and is more convenient than other methods [14,15]. The first work was reported by Bahr et al. [5], who investigated the hardness, modulus, and fracture behavior of solution deposited PZT thin film composites. Their results show that the hardness of PZT film composites is between 5GPa and 8GPa, slightly smaller than the hardness of PZT blocks, which is at about 9GPa; while the modulus of PZT film composites is between 130GPa and 160GPa, slightly larger than the modulus of bulk PZT blocks, which is between 60GPa and 130GPa [16,17]. As the delimitation between the PZT film and the substrate is one major problem for the reliability of PZT film devices [5], Zheng et al. [6] investigated the fracture behavior of PZT films by nanoindentation. They proposed an elastic groundsill model to assess the interfacial adhesion by using the data measured from nanoindentation technique. The calculated results show that the stress intensity factors for mode I and mode II crack are 0.4~1.6MPam^1/2^ and 0.6~2.2 MPam^1/2^, respectively. Later, the biaxial modulus E/(1-ν^2^) of PZT films was investigated by Delobelle et al. using nanoindentation under the continuous contact stiffness measurement procedure [7]. As the mechanical properties of PZT films are crystallographic dependent, Delobelle et al. [8] investigated the modulus and hardness of PZT films with different crystallographic orientations, and found the following relationship: E_(110)_<E_(111)_<E_(001)_ and H_(110)_<H_(111)_<H_(001)_. Here E and H mean modulus and hardness, respectively. The subscript means the crystallographic orientation of the tested PZT films. Additionally, as PZT films are normally very thin and the measured results are inevitably influenced by the deposition substrate [18,19]. Therefore, by exploring a model proposed by Zhou and Prorok [20,21], Liu et al. [9] investigated the influence of film orientation and structural layer type on the Young’s modulus of PZT films. They reported that substrate has great influence on the mechanical property of PZT films, and the orientation dependent modulus relationship E_(110)_<E_(111)_<E_(001)_ is independent on the substrate materials.

In some applications, PZT films may be exposed to high temperature due to the evolution of environment temperature or self-heating [22]. Previous investigations on ferroelectric blocks demonstrated that the mechanical property of ferroelectrics are temperature dependent due to the evolution of spontaneous strain [23–25], and high temperature may also cause performance degradation or ever failure of ferroelectric devices [23–25]. However, the mechanical property of PZT films at high temperature has not been characterized yet. Therefore, it is pertinent to understand the mechanical property of PZT films at high temperature aiming to optimize its further applications. In this article, by exploring nanoindentation technique with high temperature testing mode, the load-depth curves of an unpoled PZT film composite were tested with loading amplitude and testing temperature of 10mN and 380°C, respectively. The reduce modulus and hardness were firstly calculated by using the Oliver-Pharr method [13]. Then the effect of substrate on the measured true modulus and hardness was discussed. In addition, the influence of residual stress on the indentation behavior of PZT film composite was also investigated by measuring its load-depth curves with pre-load strains.

## Specimen and Experiment

**Fig 1:**
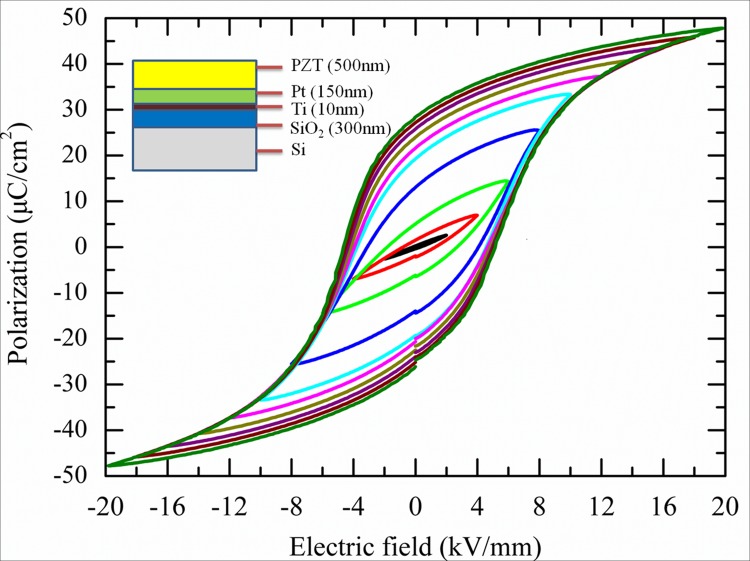
The electric field vs. polarization curves of PZT film as a function of electric field amplitude. The inset figure illustrates the inhomogeneous structure of the tested PZT film composite.

This investigation was performed on a commercial available PZT film composite provided by Inostek Inc. (South Korea). It was prepared by traditional sol-gel method on Pt(150nm)/Ti(10nm)/SiO_2_(300nm)/Si(<100> P type) substrate with thickness of 500nm (Fig. 1) [26,27]. As the texture of Pt is (111), the texture of the PZT film is (111) also due to the fact that the activation energy needed for PZT nucleation is the lowest on lattice matched substrate [27]. The Zr/Ti ratio of the material is 53/47, and thus according to the previous report [27], its crystal structure is in the vicinity of morphtropic boundary in the tetragonal range. Its coercive electric fields and remnant polarization are 4.8kV/mm and 28μC/cm^2^, respectively. Fig. 1 shows its electric field vs. polarization curves under cyclic electric fields loading with different amplitude measured by Sawyer-Tower circuit [2]. It can be seen that the switchable polarizations increase with the increasing of electric field amplitude, indicating that more domains can be switched under large electric fields.

Nanoindentation tests were performed using Hysitron Tribo Indenter (TI950) with high temperature testing mode (up to 400°C). The specimen was heated to the target operating temperature by a resistive element. Circulating water was explored to prevent damage to the unprotected components and to reduce the instability of the sensitive electronics. As the load frame is insensitive in the testing temperature range, and the diamond tip (here a typical Berkovich tip with radius of around 120nm was used) was fixed on a specific ceramic composite shaft with low thermal conductivity [28], the effect of temperature on the compliance of the equipment is negligible [29].

During testing, the specimen was firstly heated to the selected temperature. A period of 10 minutes to 30 minutes was waited to reach to thermal equilibrium of the specimen. The indentation tip was then engaged in contact with the specimen surface. Before testing was performed, the tip remained in contact with the specimen for about 1 hour to equilibrate the temperature gradient between the specimen and the indentation probe in order to reduce thermal drift [30].

## Results and Discussions

### Load-depth curves

**Fig 2:**
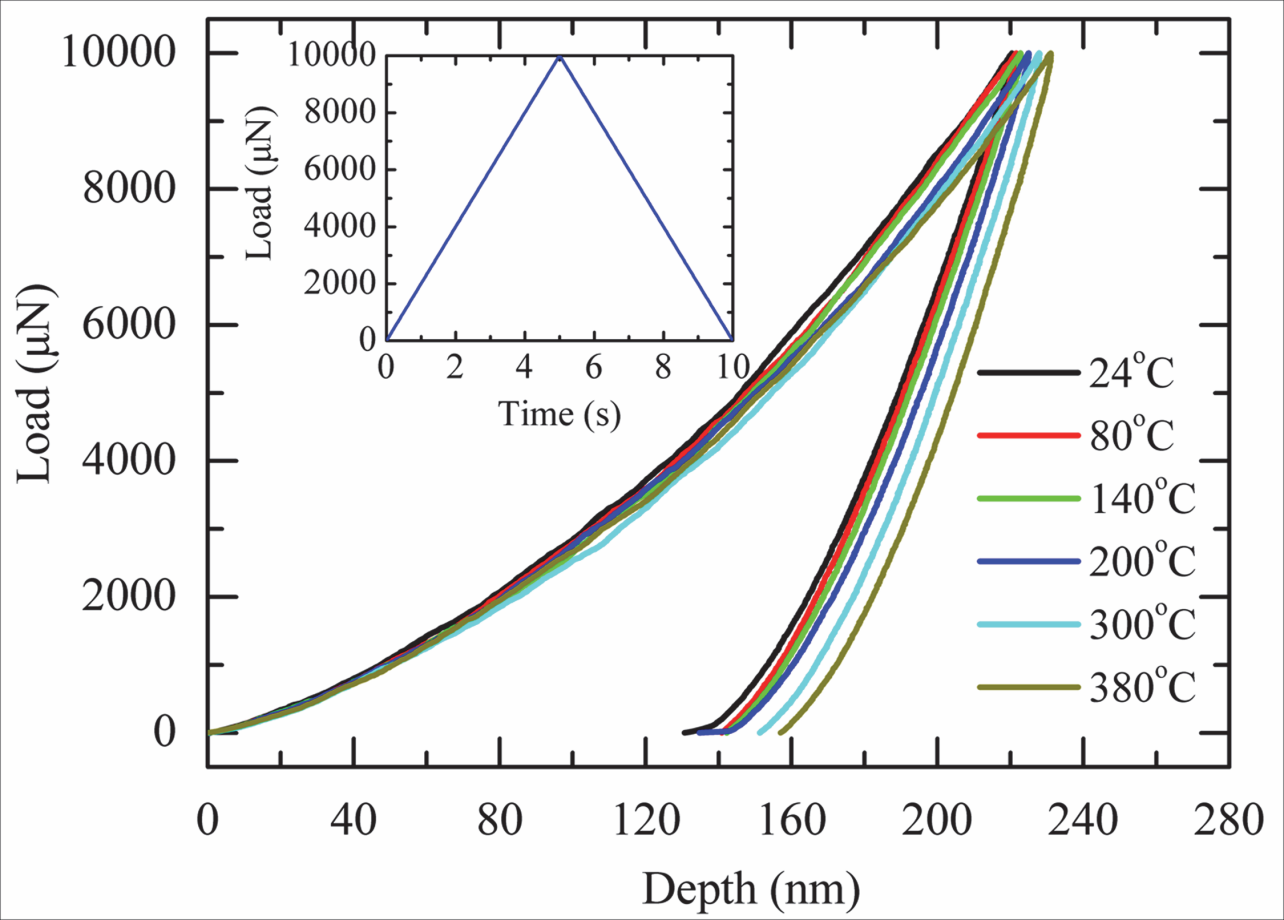
The load-depth curves of the PZT film composite as a function of temperature.

The load-depth curves of the PZT film composite as a function of temperature were firstly measured. Standard triangular waveform loading with loading rate of 2mN/s was used during testing. Five indentations were conducted at each temperature, and the measured results showed very good repeatability. Fig. 2 shows five selected load-depth curves measured at different temperature. It can be seen that the maximum indentation depth increases gradually from 220nm to 231nm when the temperature elevates from 24°C to 380°C. Significantly pop-in events were not observed during testing, indicating dislocations were activated gradually in PZT film and sudden cracking did not happen [31].

It should be noted that the deformation behavior of PZT film composite at high temperature is different from the behavior of PZT blocks during compression. Previous investigations on PZT blocks showed that their deformation ability decreases with increasing temperature [23–25]. This phenomenon was attributed to the decreasing of spontaneous strain, namely c/a ratio, at high temperature [23–25]. In addition, dislocations were not observed in PZT blocks during compression. While during nanoindentation, the indentation depth are thought to be mainly caused by dislocations [32–37], and the effect of domain reorientation on the indentation depth is limited. Since the dislocation initiation became easier with the help of thermal activation at high temperature [38], the indentation depth increases with increasing temperature.

**Fig 3:**
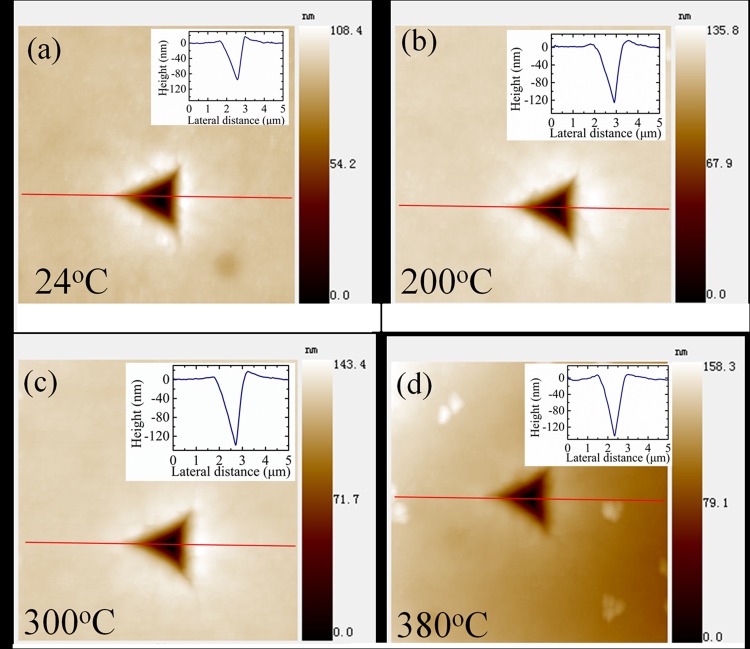
Topography micrographs of indentation impressions at 24°C, 200°C, 300°C, and 380°C. The cross-section curves of the indentation impressions were also plotted as inset figures.

The topography micrographs of the indentation impressions were scanned after indentation. Fig. 3 shows the scanned results at 24°C, 200°C, 300°C and 380°C. The cross-section curves of the topography micrographs were also plotted in Fig. 3. It can be seen that at all temperature, slight pile-up was observed near the indent impression but no apparent cracks were observed, which means that the indentation load amplitude of 10mN is not large enough to induce cracks in the tested PZT film composite. Further, the residual indentation depth (${\text{h}}_{\text{r}}^{\text{'}}$) measured by the cross-section curves increases at elevated temperature, but all of them are slightly smaller than the remnant depth (h_r_) measured by the load-depth curves. Additionally, the difference between h_r_ and ${\text{h}}_{\text{r}}^{\text{'}}$, namely ${\text{h}}_{\text{r}}-{\text{h}}_{\text{r}}^{\text{'}}$ decreases with increasing temperature, indicating some indentation depths were recovered after unloading and the recovery depth decreases with increasing temperature.

After force unloading, the recovery of indentation depth is thought coming from two contributions. One part is caused by the recovery of the elastic deformation, which should keep nearly constant at all tested temperature; the other part is induced by the back-switched domains after unloading. Previous investigations demonstrated that with the increasing of temperature, the spontaneous strain of PZT materials decreases. Consequently, the recovery depth induced by back-switch domains would decrease with the increasing of temperature. As a result, the total recovery depth decreases with increasing temperature.

### Modulus and Hardness

#### A Reduce modulus and Hardness of the tested PZT film composite measured by partial unloading method

**Fig 4:**
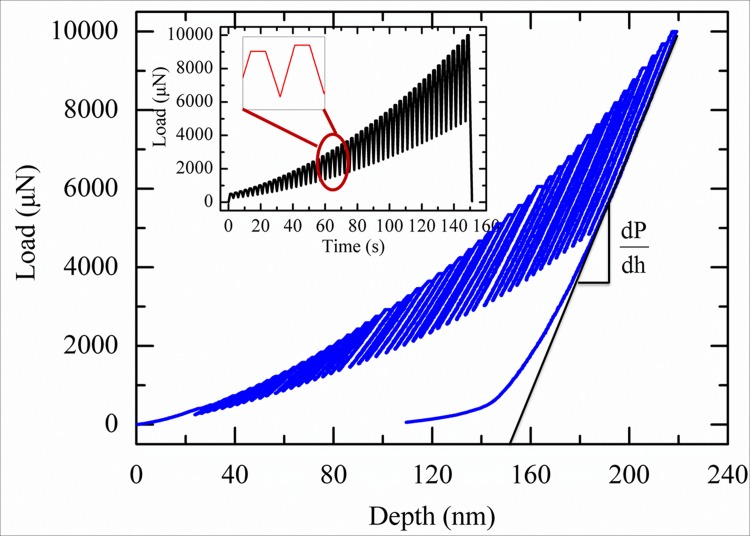
The load-depth curve of PZT film composite under partial unloading function at 24°C.

The modulus and hardness of the PZT film composite as a function of loading amplitude and temperature were then measured throughout standard partial unloading method (The inset figure in Fig. 3 illustrates the loading function). Fig. 4 shows the represented load-depth curve of the PZT film composite at 24°C. The reduce modulus and hardness were then calculated by the typical Oliver-Pharr method [13]. The reduced modulus was calculated by equation:

$$E_{r}=\frac{S\sqrt{\pi }}{2\sqrt{A}}$$ (1)

The hardness was calculated by equation:

$$H=\frac{P_{\text{max}}}{A}$$ (2)

Where *E_r_* is the reduce modulus; *S* is the contact stiffness, calculated by $\frac{dP}{dh}$, as shown in Fig. 3; *A* is the contact area. The contact area A is calculated by $A=C_{0}h_{c}^{2}+C_{1}h_{c}+C_{2}h_{c}^{1/2}+C_{3}h_{c}^{1/4}+C_{4}h_{c}^{1/8}+C_{5}h_{c}^{1/16}$, in which *C*_0_ is equal to 24.5 for Berkovich indenter tip; *C*_1_ through *C*_5_ were got throughout typical calibration method proposed by Oliver and Pharr by using fuse silicon as standard material. It should be noted that the contact area A calculated at room temperature is also applicable for high temperature testing. Although the diamond tip will expansion at a rate of 5×10^−7^/°C [39], its influence on the tip area function is limited because thermal expansion is a geometric self-similar process [29].

**Fig 5:**
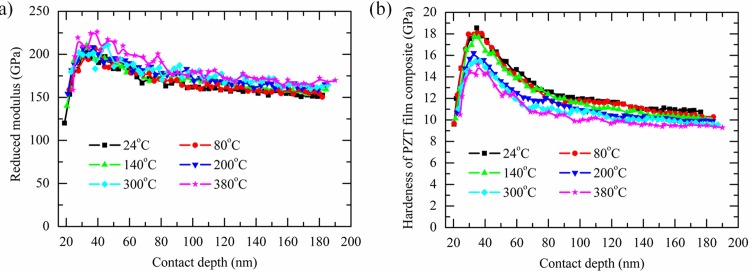
Reduce modulus vs. contact depth curves (A), and hardness vs. contact depth curves of PZT film composite measured at different temperature (B).

Fig. 5A and 5B show the calculated reduce modulus and hardness at different temperature, respectively. It can be seen that with the increasing of temperature, the reduce modulus gradually increases but the hardness decreases at the same contact depth. When the temperature keeps constant, both the measured reduce modulus and hardness first increase and then decease with the increasing of the contact depth.

It should be pointed that the measured reduce modulus is a composite elastic parameter including the properties of the PZT film composite and the diamond indenter tip, which is expressed by [13]:

$$\frac{1}{E_{r}}=\frac{1-\nu_{d}^{2}}{E_{d}}+\frac{1-\nu_{s}^{2}}{E_{s}}$$ (3)

Where E_d_ and E_s_ mean the modulus of diamond and PZT film composite, respectively; *v_d_* and *v_d_* are the Poisson’s ratio of diamond and the PZT film, which are 0.07 and 0.3, respectively. Then the modulus of the PZT film composite can be calculated by:

$$E_{s}=\frac{E_{d}E_{r}\left(1-\nu_{s}^{2}\right)}{E_{d}-E_{r}\left(1-\nu_{d}^{2}\right)}$$ (4)

Additionally, it is known that the Young’s modulus of diamond decreases at elevated temperature, and can be expressed by [40]:

$$E_{d}=E_{d}^{RT}\left[1+c\left(T-293\right)\right]$$ (5)

Where $E_{d}^{RT}$ is the modulus of diamond at room temperature. C is a constant with value of −1.027×10^−4^/°C. As the Poisson’s ratio is temperature independent, then by combination equation 4 and 5, the true modulus of PZT film composite at different temperature can be calculated by equation 4.

**Fig 6:**
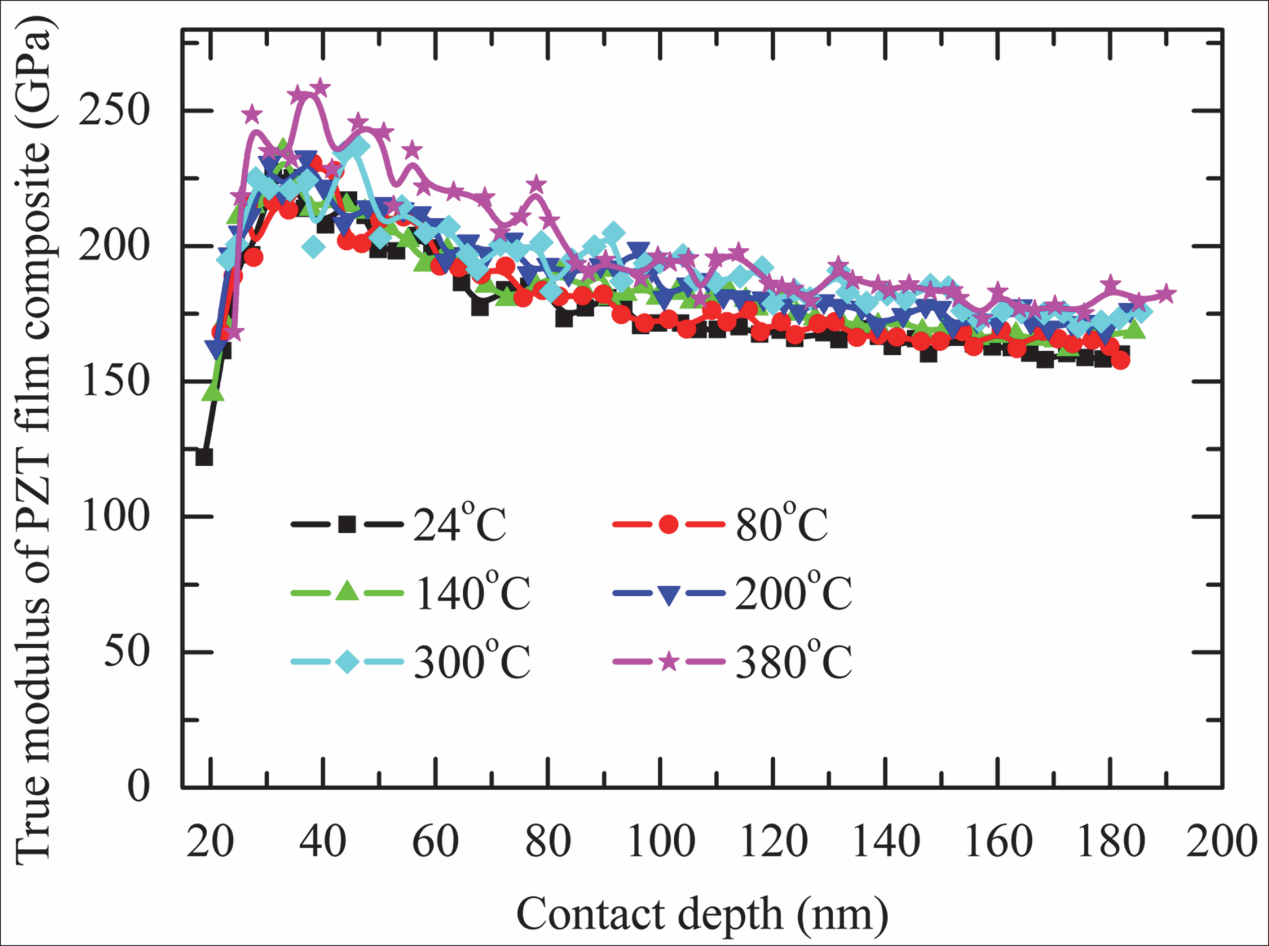
The true modulus of PZT film composite vs. contact depth at different temperature.

Fig. 6 shows the calculated true modulus of PZT film composite by equation 4 at different temperature. Results show that at constant contact depth, its true modulus increases with the increasing of temperature.

#### B Calculating the true modulus and hardness of PZT film by taking the substrate effect into consideration.

As the PZT film is deposited on Pt/Ti/SiO_2_/Si substrate, determining its true mechanical properties is complex because of the influence of substrate on the measured results [26,27]. Several models have been developed to decouple the effect of substrate aiming to assess the true modulus of the film tested. Among them the discontinuous elastic interface transfer model proposed by Zhou and Prorok [20,21] fits the experimental results better than the continuous elastic model proposed by Gao et al. [42] and Doerner et al. [41]. Based on their assumption, the modulus of the composite, the modulus of film, and the modulus of substrate can be related by:

$$\frac{1}{E_{c}}=\frac{1}{E_{f}}\left(1-\Phi_{s}\right)\cdot {\left(\frac{E_{f}}{E_{s}}\right)}^{0.1}+\frac{1}{E_{s}}\Phi_{f}$$ (6)

Where E_c_ is the modulus of a film composite; E_f_ means the modulus of the film; E_s_ represents the modulus of the substrate; Φ*_f_* and Φ*_s_* represent the weighting factors account for the effects of the film on the substrate and the substrate on the film, respectively, and are expressed as:

$$\Phi_{f}=e^{-\alpha_{f}\left(t/h\right)}$$ (7)

$$\Phi_{s}=e^{-\alpha_{s}\left(t/h\right)}$$ (8)

Here α*_f_* and α*_s_* ratio the film and substrate, which are 0.38 and 0.3 [9], respectively; t is the thickness of the film; h means the maximum indentation depth.

**Fig 7:**
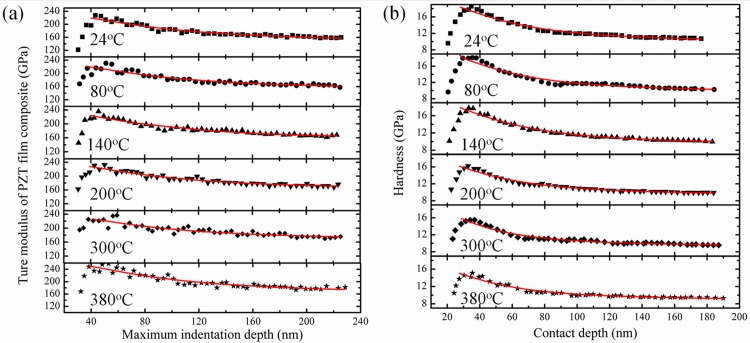
True modulus of the PZT film composite vs. maximum indentation depth curves at different temperature (a), and hardness vs. contact depth curves of the PZT film composite at different temperature. The black dots represent the experimental results (b). The red lines in Fig. 7A and Fig. 7B represent the fitting results by equation 6 and equation 9, respectively.

Then by fitting equation 6 with the experimental results, the true modulus of the PZT film and the substrate can be assessed. It should be noted that when the maximum indentation depth is less one third of the radius modulus of the indenter tip, the measured modulus and hardness is typically not valid [13]. Therefore, only the data with the maximum indentation depth larger than 40nm was used during fitting. Fig. 7A shows the fitting results.

Additionally, the measured hardness will also be influenced by the substrate. In this article, the model proposed by Korsunsky et al. [18] was used to calculate the true hardness of the PZT film. According to their model, the hardness of the film composite is given by:

$$H_{c}=H_{s}+\frac{H_{f}-H_{s}}{1+k\beta^{2}}$$ (9)

Where H_f_ and H_s_ are hardness of film and substrate, respectively. k is a fitting parameters, and β is the normalized depth calculated by h_c_/t; here t means the thickness of the PZT film. Similarly, only the data with the maximum indentation depth larger than 40nm was used to fit hardness. Fig. 7B shows the fitting result.

The true modulus and true hardness of the PZT film calculated by equation 6 and equitation 9 are listed in table 1. The true modulus at room temperature is 228GPa, larger than the previous reported values, which is at about 150~170GPa [5,9]. Additionally, it increases from 228GPa to 239GPa gradually between 24°C and 300°C. When the temperature reaches to 380°C, it quickly increases to 257GPa. While for the hardness, the calculated result is 19.8GPa at room temperature, which is larger than the previous reported value of 5~8GPa [5]. With increasing of temperature, it decreases gradually from 19.8GPa to 16.4GPa. The mechanism of why the measured modulus and hardness are larger than previous reports are not clearly yet. Thus further work is still needed to get the exact mechanical parameters of PZT films.

**Table 1:** The calculated Young’s modulus and Hardness of the PZT film and the substrate at different temperature.

Temperature		24°C	80°C	140°C	200°C	300°C	380°C
True modulus (GPa)	PZT film	228	229	232	233	239	257
	Substrate	134	139	144	149	147	146
True Hardness (GPa)	PZT film	19.8	19.6	19.2	18	17.3	16.4
	Substrate	9.7	9.5	9.2	9.1	9.0	8.7

#### C The effect of misfit strain between the PZT film and the substrate on the mechanical property of PZT film composite

It is known that residual stress exists in PZT film composite due to misfit strain between PZT film and substrate when it is cooled through the Curie temperature [43,44]. Additionally, previous investigations showed that pre-load stresses have great influence on the nanoindentation behavior of elastic-plastic materials: the indentation depth decreases at pre-compression state but increases at pre-tension state [45]. Moreover, it is known that the spontaneous strain, name c/a ratio, will change with evolution of temperature, which will influence the residual stress in PZT films [23]. Therefore, to understand to what extent the temperature can influence the nanoindentation behavior of PZT films, the effect of residual stress on its mechanical property should be understood.

**Fig 8:**
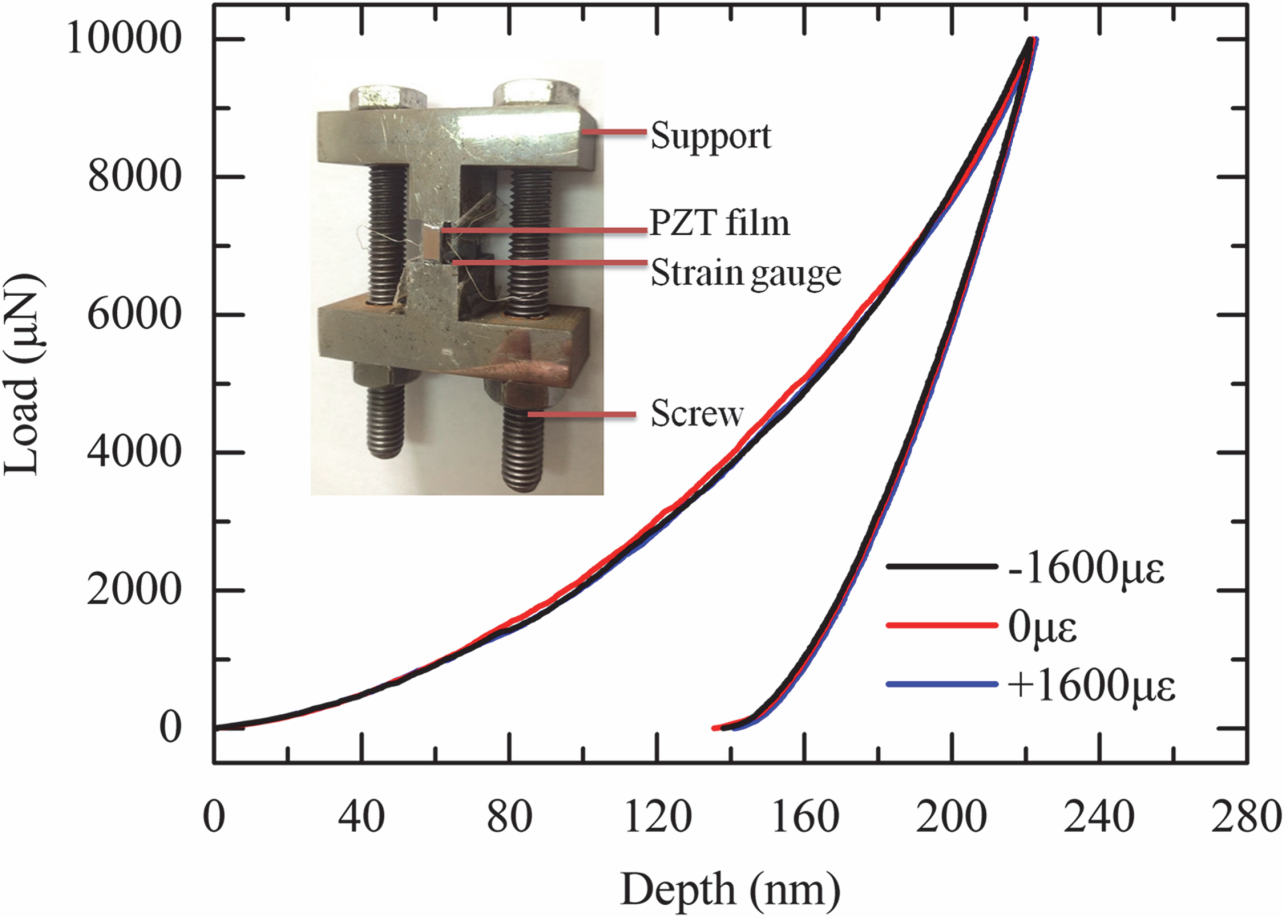
The load-depth curves of the PZT film composite at different pre-load states. The inset figure shows the loading stage.

We designed a simple steel (Q235) loading stage to apply pre-load stress to PZT film composite (Fig. 8). PZT film composite was pasted on its surface. Two strain gauges were glued on two opposite lateral surfaces of the loading part, and one strain gauge was pasted on the surface of the PZT film composite to monitor the applied strains and the portion of strains can be transferred from the loading stage to the PZT film. Two screw bolts were used to apply stress to the stage. During loading, it is found only 80% of the applied strains can be transferred from the loading stage to PZT film.

Then nanoindentation was performed on the pre-loaded PZT film composite. Fig. 8 shows its load-depth curves at 1600 pre-load tensile strain state, 1600 pre-load compressive strain, and strain-free state. It can be seen that the measured load-depth curves are nearly overlapped with each other, indicating that pre-load strains hardly have any effects on the indentation behavior of PZT film. Such phenomenon is difference from the previously reported results in elastic-plastic materials by Tsui: the indentation depth decreases at pre-compression state but increases at pre-tension state [45]. This difference can be qualitatively explained by the physical difference beneath the indenter. When nanoindentation was performed in elastic-plastic materials, the plastic zone will change beneath the indenter under the influence of pre-load stresses due to dislocation evolution [45]. However, for PZT films, the dislocations were concentrated near the surface of the indenter due to the fact that PZT film is brittle material and dislocation motion in it is nearly impossible. As a result, it can be concluded that the influence of pre-load stress on the indentation behavior of PZT film is negligible.

#### D Discussions

Based on the above analysis, it can be concluded that for the tested PZT film, its modulus increases, but hardness decreases at elevated temperature, and residual stress hardly have any influences on its mechanical behavior. Thus the factors that influence the mechanical property of PZT film can be attributed to domain rearrangement and dislocation evolution beneath the indenter during loading and unloading.

It has been reported that in BaTiO_3_ crystal, both dislocation initiation and domain switching happened during indentation [32–37,46]. Since domain switching can also induce deformation in ferroelectrics, a part of the indentation depth is thought to be caused by domain reorientation during loading [47]. Such behavior was also verified by our recent work performed in BaTiO_3_ crystal [unpublished work]. Our results show that for BaTiO_3_ crystal, the measured indentation depth at room temperature is larger than the indentation depth measured at high temperature (above the Curie temperature). However, for PZT films, the indentation depth induced by domain switching should be limited. Firstly, the coercive stress needed to cause domain switching in PZT films is significantly larger than that in BaTiO_3_ single crystal [23–25,48]. What’s more, the domain wall motion ability in PZT film is influenced by its small grain size [49]. As a result, most indentation depth in PZT film should be mainly caused by dislocations. As aforementioned, the dislocation initiation would become easier with the help of thermal activation at high temperature [38]. So the indentation depth increases with the increasing of temperature in PZT film.

During unloading, some switched domains, first due to their easier mobility, and on others due to the constraint coming from their neighbor domains, switched back during unloading. A part of the indentation depth recovery was thought to be caused by these back-switched domains. As the spontaneous strain decreases at elevated temperature, the portion of indentation depth caused by back-domain switching decreases with the increasing of temperature. Thus, the modulus of PZT film increases at high temperature. When the temperature elevates from 300°C to 380°C, the PZT film changes from ferroelectric phase to paraelectric phase and there are no domains switch back during unloading in 380°C. As a result, a significant increasing of modulus was observed from 300°C to 380°C (Fig. 6 and Fig. 7A).

## Summary and Conclusion

In summary, the mechanical property of an unpoled PZT film composite as a function of temperature (24°C to 380°C) was investigated by using nanoindentation technique. Its load-depth curves at different temperature were measured. Its reduce modulus and hardness were calculated by the typical Oliver-Pharr method. Then the true modulus and hardness of PZT film were assessed by decoupling the influence of substrate using methods proposed by Zhou et al. and Korsunsky et al., respectively. The influence of residual stress on the indentation behavior of PZT film composite was also investigated by measuring its load-depth curves with different pre-load strains. The following conclusions were got:

1. The indentation depth of PZT film increases with the increasing of temperature. As a result, its hardness decreases with the increasing of temperature. Such behavior is thought to be caused by the decreasing of the critical stress needed to excite dislocation in PZT film at high temperature.
2. The Young’s modulus of PZT film increases with the increasing of temperature. This phenomenon is attributed to the decreasing of the recoverable indentation depth induced by back-switched domains at high temperature.
3. Residual stresses hardly have any influences on the mechanical behavior of PZT film composites.

These results and conclusions are thought to be helpful for designing devices made of PZT films.
